# High Frequency Needle Ultrasonic Transducers Based on Lead-Free Co Doped Na_0.5_Bi_4.5_Ti_4_O_15_ Piezo-Ceramics

**DOI:** 10.3390/mi9060291

**Published:** 2018-06-10

**Authors:** Chunlong Fei, Tianlong Zhao, Danfeng Wang, Yi Quan, Pengfei Lin, Di Li, Yintang Yang, Jianzheng Cheng, Chunlei Wang, Chunming Wang, Qifa Zhou

**Affiliations:** 1 School of Microelectronics, Xidian University, Xi’an 740071, China; clfei@xidian.edu.cn (C.F.); zhaotl@xidian.edu.cn (T.Z.); jikedandan@163.com (D.W.); pflinxidian@163.com (P.L.); lidi2004@126.com (D.L.); yangyt@xidian.edu.cn (Y.Y.); 2 School of Electronic and Electrical Engineering, Wuhan Textile University, Wuhan 430200, China; jzcheng@wtu.edu.cn; 3 Electronic Materials Research Laboratory, Key Laboratory of the Ministry of Education & International Center for Dielectric Research, Xi’an Jiaotong University, Xi’an 740049, China; quanyi@stu.xjtu.edu.cn; 4 School of Physics, State Key Laboratory of Crystal Materials, Shandong University, Jinan 250100, China; wangcl@sdu.edu.cn; 5 Department of Ophthalmology and Biomedical Engineeing, University of Southern California, Los Angeles, CA 90089-1111, USA

**Keywords:** lead-free piezoelectric materials, high frequency ultrasonic transducer, needle-type, high spatial resolution

## Abstract

This paper describes the design, fabrication, and characterization of tightly focused (ƒ-number close to 1) high frequency needle-type transducers based on lead-free Na_0.5_Bi_4.5_Ti_3.975_Co_0.025_O_15_ (NBT-Co) piezo-ceramics. The NBT-Co ceramics, are fabricated through solid-state reactions, have a piezoelectric coefficient *d*_33_ of 32 pC/N, and an electromechanical coupling factor *k_t_* of 35.3%. The high Curie temperature (670 °C) indicates a wide working temperature range. Characterization results show a center frequency of 70.4 MHz and a −6 dB bandwidth of 52.7%. Lateral resolution of 29.8 μm was achieved by scanning a 10 μm tungsten wire target, and axial resolution of 20.8 μm was calculated from the full width at half maximum (FWHM) of the pulse length of the echo. This lead-free ultrasonic transducer has potential applications in high resolution biological imaging.

## 1. Introduction

Tightly focused high frequency ultrasonic transducers have many clinical applications, ranging from imaging the skin and eye to small animal imaging, because of their improved image resolution [1,2,3,4]. The improvement of axial resolution is due to a reduction in wavelength, and thus pulse duration, for a fixed number of cycles per pulse. The improvement of on-focus lateral resolution is due to the small ƒ-number, defined as the ratio of the focal distance to the spatial dimension of the transducer, of the tightly focused transducer and the wavelength. As the key component of ultrasonic transducers, piezoelectric materials such as zinc oxide (ZnO) and aluminum nitride (AlN) piezoelectric films [5,6], lead oxide based ferroelectrics, especially the lead zirconate titanate (PZT) system, lead niobiumzine zirconate titanate (PMN-PT) crystal [7,8,9], lithium niobate (LiNbO_3_) single crystal, [4] and lead-free piezoelectric ceramics [10,11,12] have been investigated extensively. Among them, due to their environmental friendliness, simple preparation methods, and relatively low cost, lead-free piezoelectric ceramics have attracted significant interest.

Lead-free Na_0.5_Bi_4.5_Ti_4_O_15_ (abbreviated as NBT) based materials have been studied recently for their interesting properties, such as high Curie temperature, low dielectric loss, and reasonable piezoelectric behaviors [13,14,15]. However, the piezoelectric performance of pure NBT ceramics is quite weak; the value of piezoelectric coefficient *d*_33_ is 16 pC/N [16]. Researchers have made many attempts, such as A- or B-site modification and grain orientation techniques, to overcome these shortcomings and improve the piezoelectric and ferroelectric properties of NBT ceramics [13,17,18]. In these studies, the B-site acceptor modification improved the piezoelectricity of NBT ceramics significantly [15]. For example, the value of *d*_33_ of Co-doped NBT (NBT-Co) piezoelectric ceramics is over 30 pC/N, which is almost twice of the value of pure NBT ceramics.

To the best of the authors’ knowledge, transducers based on NBT-based ceramics for high frequency ultrasound applications have rarely been reported. In this paper, NBT-Co ceramics were prepared and systematically investigated. A tightly focused needle-type ultrasonic transducer with center frequency of 70 MHz was designed and fabricated based on the performance of the NBT-Co ceramics. The electrical and acoustic properties of the transducer were investigated in detail, which demonstrated the great potential of this lead-free transducer for application in high resolution biological and medical imaging.

## 2. Fabrication and Characterization of Co-Doped Na_0.5_Bi_4.5_Ti_4_O_15_ Ceramics

### 2.1. Fabrication

A conventional mixed-oxide technique was used to prepare sodium-potassium bismuth titanate (NBT-Co) piezoelectric ceramics. Analytical grade Na_2_CO_3_ (99.9%), Bi_2_O_3_ (99.9%), TiO_2_ (99.9%), and Co_2_O_3_ (99.9%) were selected as starting materials. The composition investigated in the present work is Na_0.5_Bi_4.5_Ti_3.975_Co_0.025_O_15_ (abbreviated as NBT-Co). The mixture was wet milled in polyethylene bottles with ZrO_2_ balls for 12 h in ethanol and calcined at 800 °C for 2 h. Then the mixture was milled again in the same conditions. The milled powders were dried, ground, and granulated with polyvinyl alcohol (PVA) binder, then pressed into disks at a pressure of 150 MPa. The green compacts were put into a sealed crucible fully surrounded with powder having the same composition and sintered at 1080–1100 °C for 3 h. After cooling to room temperature freely, the size of the final samples was 13 mm in diameter and 0.5 mm in thickness.

Phase structure was determined by X-ray diffraction technology with Cu*K*α_1_ (λ = 1.540596 Å) radiation (D8 Advance; Bruker AXS GMBH, Karlsruhe, Germany). Surface micromorphology of the sintered ceramics was detected by scanning electron microscopy (SEM, S-4800, Hitachi, Tokyo, Japan). The piezoelectric coefficient *d*_33_ was measured by a quasi-static *d*_33_ meter (ZJ-2, Institute of Acoustics, Academia Sinica, Taipei, Taiwan). Dielectric measurements were performed with a 4294A impedance analyzer (Agilent Technologies, Santa Clara, CA, USA). The electromechanical coupling factors (*k_p_*, *k_t_*), and frequency constants (*N_p_*, *N_t_*) were calculated according to IEEE standards [19].

### 2.2. Characterization

**Figure 1:**
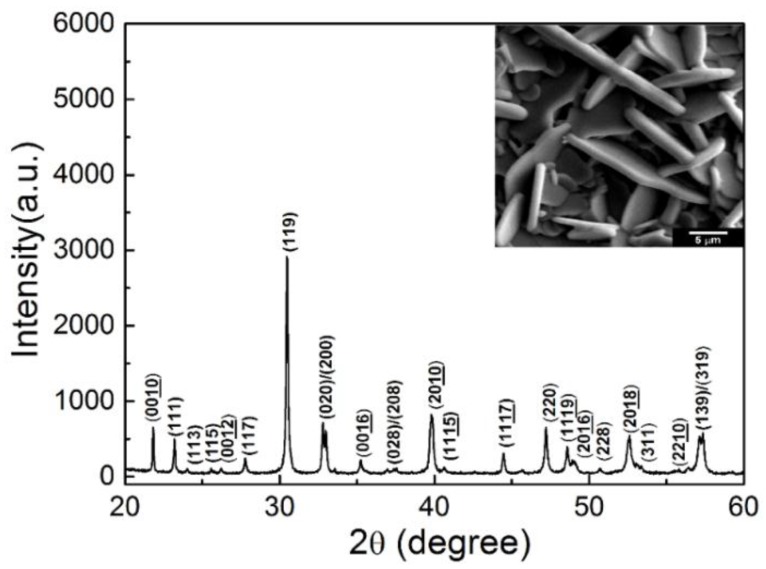
The X-ray diffraction pattern and scanning electron microscopy (SEM) image (embedded) of the Co-doped Na_0.5_Bi_4.5_Ti_4_O_15_ (NBT-Co) sample.

Figure 1 shows the X-ray diffraction pattern of the NBT-Co piezoelectric ceramic powder. The diffraction pattern is in agreement with the diffraction data for NBT ceramics, indicating that the Co doping does not change the crystal structure of NBT ceramics. The surface of the NBT-Co piezoelectric ceramics is shown in the inset scanning electron microscopy (SEM) image. As shown, the sample has a dense structure and plate-like morphology, corresponding with the high anisotropy of NBT-Co ceramics.

Detailed room temperature electrical properties of the NBT-Co polycrystalline piezoelectric ceramics are in Table 1. The piezoelectric coefficient *d*_33_ of NBT-Co ceramics was found to be 32 pC/N, with a large enhancement of double that of pure NBT ceramics. In addition, the relative permittivity *ɛ* and dielectric loss tan *δ* at 1 MHz are 148 and 0.26%, respectively. Furthermore, the planar electromechanical coupling factor (*k_p_*) and thickness electromechanical coupling factor (*k_t_*) were calculated according to the resonance method and found to be 5.2% and 35.3%, respectively. In addition, the planar frequency constant (*N_p_*) and thickness frequency constant (*N_t_*) makes little difference and were found to be 2320 and 2280, respectively.

**Figure 2:**
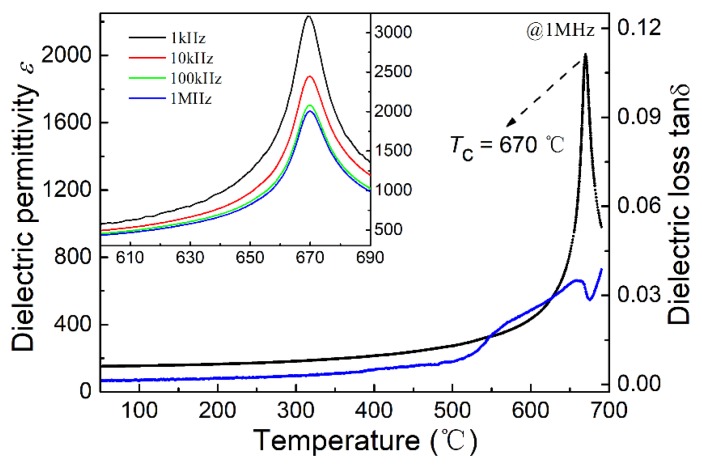
The relative permittivity (black curve) and dielectric loss (blue curve) of the Co-doped Na_0.5_Bi_4.5_Ti_4_O_15_ (NBT-Co) ceramic measured at 1 MHz and other frequencies (embedded) as a function of temperature.

Figure 2 shows the relative permittivity *ε* and dielectric loss tan *δ* measured at 1 kHz, 10 kHz, 100 kHz, and 1 MHz as a function of temperature for the cobalt-modified NBT piezoelectric ceramics. The Curie temperature was found to be 670 °C and remains unchanged at all measured frequencies, indicating typical diffuse phase transition characteristics. It can be seen that the dielectric loss tan *δ* is lower than 1% even when the temperature reached 400 °C; this is important for high temperature piezoelectric applications.

## 3. Design, Fabrication, and Characterization of Tightly Focused Needle Ultrasonic Transducer

### 3.1. Transducer Design and Fabrication

**Figure 3:**
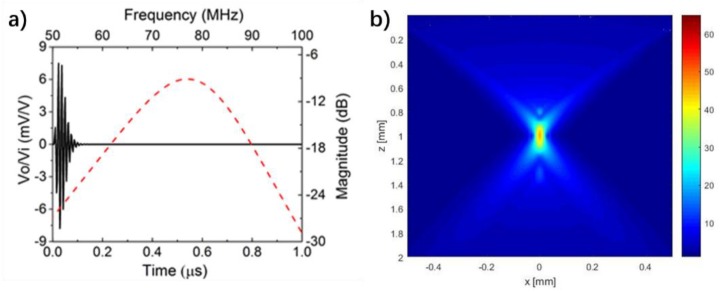
(**a**) Modelling results of Co-doped Na_0.5_Bi_4.5_Ti_4_O_15_ (NBT-Co) single element transducer from Krimholtz, Leadom, and Mettaei (KLM) model-based simulation software PiezoCAD. (**b**) Simulated intensity profile in X-Z plane of NBT-Co transducer with ƒ-number of 1.

Krimholtz, Leadom, and Mettaei (KLM) model-based simulation software PiezoCAD (Sonic Concepts, Woodinville, WA, USA) was used for transducer design. Finite element modeling software Field II was utilized to give the theoretical analysis of imaging performance by simulating the intensity profile in the X-Z plane. Key parameters of NBT-Co ceramics used for simulation are in Table 2. During the simulation, E-solder 3022 and Parylene C were selected as backing and matching materials, respectively, which is consistent with the experiment. The pulse-echo simulation results (shown in Figure 3a) show transducer design with center frequency of 76.2 MHz and −6 dB bandwidth of 31.8%; the Field II simulation results (shown in Figure 3b) show the on-focus −6 dB beam width to be less than 30 μm for NBT-Co transducer with ƒ-number = 1. All these results suggest that NBT-Co ceramics can be used for high frequency and high resolution transducer applications.

**Figure 4:**
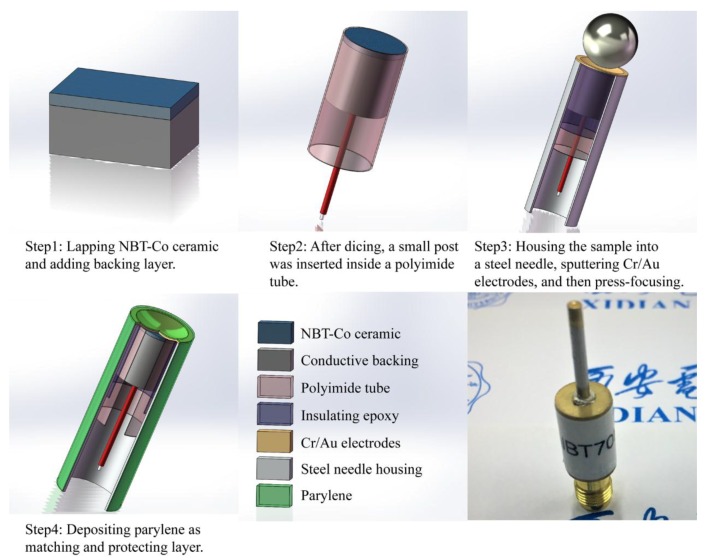
The schematic diagram and photograph of the Co-doped Na_0.5_Bi_4.5_Ti_4_O_15_ (NBT-Co) needle transducer.

Based on the prepared NBT-Co piezoelectric ceramics, a high frequency press-focused needle-type transducer was design and fabricated. The schematic diagram and photograph of the designed NBT-Co high frequency ultrasonic transducer are shown in Figure 4. Firstly, the NBT-Co ceramic was manually lapped to around 30 μm, per the design. Au (100 nm) electrodes were sputtered on one side of the NBT-Co ceramic; E-solder 3022 was then cast on this side as the backing material, which was lapped to 2 mm. The sample was diced to 0.7 × 0.7 mm^2^ posts using a dicing saw (DAD 323, Disco, Tokyo, Japan) and housed inside a polyimide tube, which provided electrical isolation for the element. The entire assembly was sealed in a steel needle. A lead wire was connected to the backing layer at one side and a SubMiniature version A (SMA) connector at the other side. Then a 100 nm Au layer was sputtered across the front surface to form the ground plane connection. The transducer was press-focused at 90 °C by a highly polished chrome/steel ball bearings with diameter of 2 mm using a set of customer designed fixtures. Finally, a PDS 2010 Labcoator (Specialty Coating Systems, Indianapolis, IN, USA) was used to vapor-deposit a ~8 μm layer of Parylene C on the front face of the transducer to serve as an acoustic matching layer.

### 3.2. Transducer Characterization

**Figure 5:**
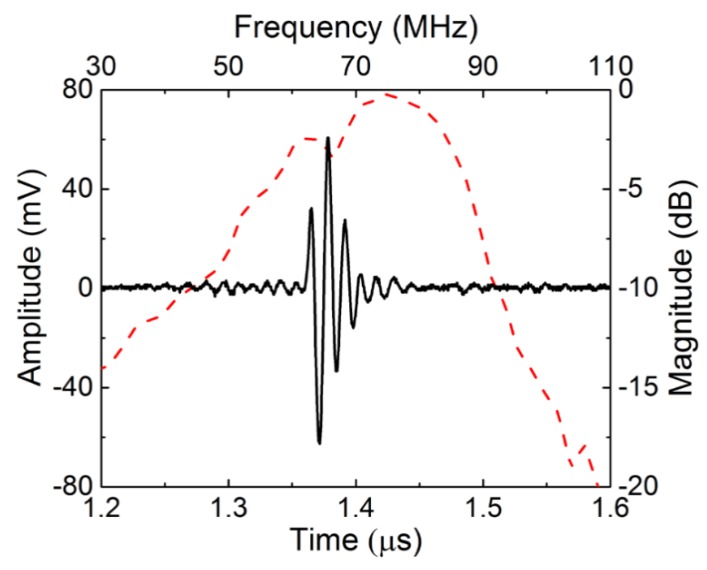
Time-domain pulse/echo response and normalized frequency spectrum of Co-doped Na_0.5_Bi_4.5_Ti_4_O_15_ (NBT-Co) transducers.

The pulse-echo response of the NBT-Co needle transducer was measured in distilled water through conventional means [4]. The pulse-echo response and frequency spectrum of the NBT-Co press-focused transducer are shown in Figure 5. The measured transducer performance is in Table 3. As can be seen, the NBT-Co transducer exhibits center frequency of 70.4 MHz and a −6 dB bandwidth of 52.7%. The ƒ-number was calculated to be 1.03. The small ƒ-number, which leads to narrow beam width, is expected to yield high lateral resolution.

**Figure 6:**
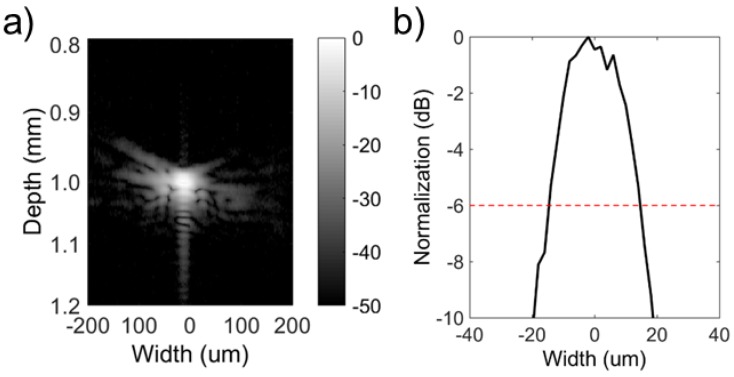
(**a**) Image of 10 μm tungsten wire target; (b) Lateral beam profile of the Co-doped Na_0.5_Bi_4.5_Ti_4_O_15_ (NBT-Co) transducers.

The lateral beam profile of the transducer was evaluated by scanning a wire phantom made of 10 μm diameter tungsten wire. The pulse intensity integral (PII) was calculated from the wire target. As shown in Figure 6, a beam width equal to 29.8 μm was obtained by the NBT-Co needle transducer in detecting a spatial point target at full width at half maximum (FWHM, −6 dB). In addition, the axial resolution was calculated from the FWHM of the pulse length of the echo to be 20.8 μm.

## 4. Conclusions

Lead-free NBT-Co ceramics were fabricated and investigated systematically. Based on the prepared NBT-Co ceramics, a high frequency and small ƒ-number transducer was designed and fabricated. The fabricated transducer has center frequency of 70.4 MHz, −6 dB bandwidth of 52.7%, and ƒ-number close to 1. Axial resolution of 20.8 μm was calculated from the FWHM of the pulse length of the echo, and lateral resolution of 29.8 μm was achieved using tungsten wire imaging. These results illustrate that lead-free NBT-Co ceramics have great potential for high frequency ultrasonic applications.

## Figures and Tables

**Table 1:** Electrical properties of Co-doped Na_0.5_Bi_4.5_Ti_4_O_15_ (NBT-Co) ceramics.

T_c_ (°C)	*ε*_33_^T^	Tan *δ* (%)	*d*_33_ (pC/N)	*k_p_* (%)	*k_t_* (%)	*N_p_* (Hz·m)	*N_t_* (Hz·m)
670	148	0.26	32	5.2	35.3	2320	2280

**Table 2:** Parameters of Co-doped Na_0.5_Bi_4.5_Ti_4_O_15_ (NBT-Co) ceramics used for PiezoCAD modeling.

Property	NBT-Co
Longitudinal velocity *υ*	4600 m/s
Density *ρ*	6500 kg/m^3^
Acoustic impedance *Z*	29.9 MRayl
Clamped relative dielectric constant *ε_r_*	20
Dielectric loss tan *δ*	0.0026
Thickness electromechanical coupling *k_t_*	0.353
Piezoelectric coefficient *d*_33_	32 pC/N

**Table 3:** Measured Co-doped Na_0.5_Bi_4.5_Ti_4_O_15_ (NBT-Co) transducer performance.

Property	NBT-Co Transducer
Center frequency (MHz)	70.4
Peak to peak Voltage (mV) @ 0 dB gain	123
−6dB Bandwidth	52.7%
Focus depth (mm)	1.02
ƒ-number	1.03
